# Supplementary material for: Non-Typeable Haemophilus influenzae Invade Choroid Plexus Epithelial Cells in a Polar Fashion
Source: Int J Mol Sci. 2020 Aug 10;21(16):5739. doi: 10.3390/ijms21165739 (PMC7461124; doi:10.3390/ijms21165739)
Supplement: Supplementary file 1 [file ijms-21-05739-s001.pdf]

S1

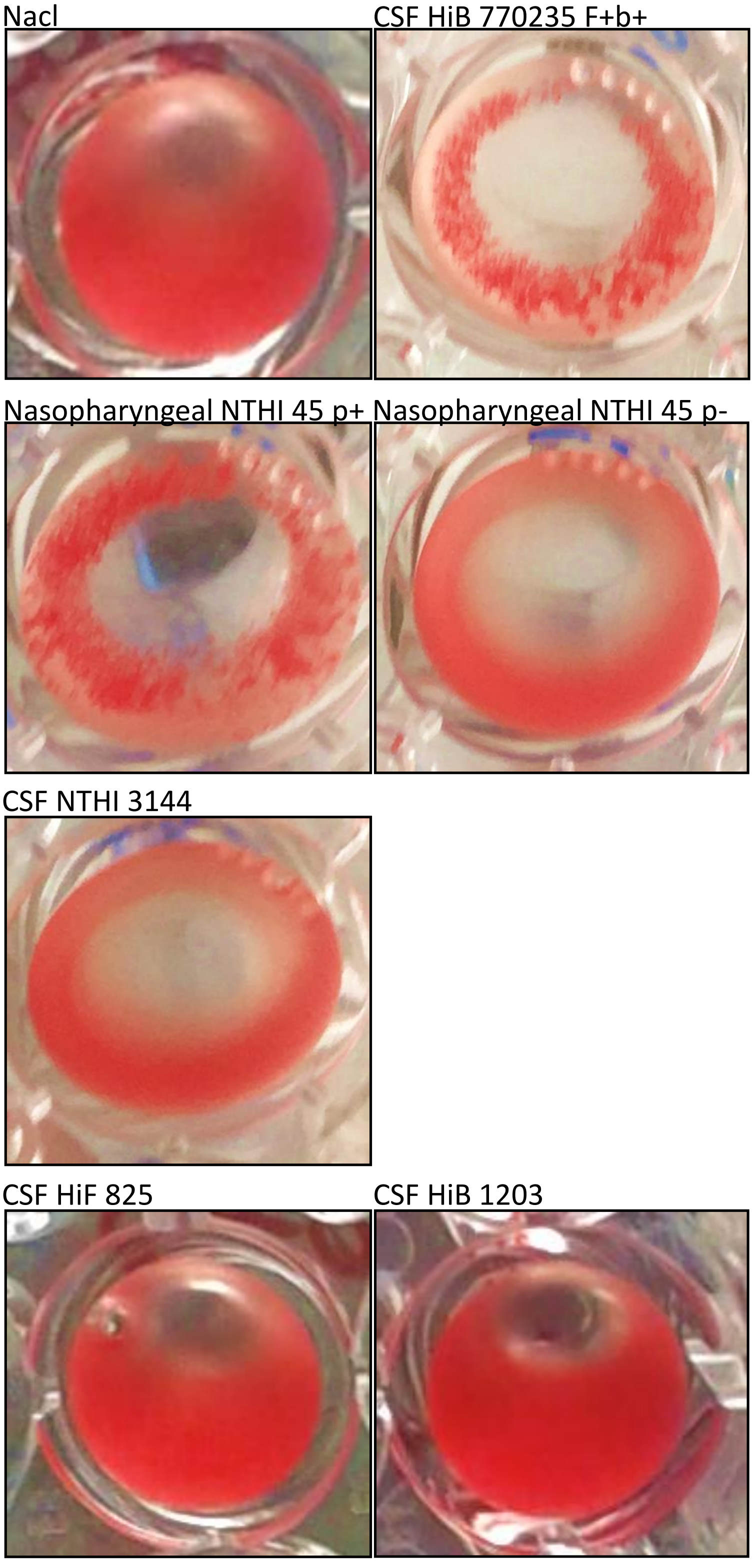

Figure S1: Hemagglutination of erythrocytes of fresh blood indicating the presence of hemagglutinating fimbriae. Cells were mixed with the indicated strains suspended in NaCl 0,9 % and hemagglutination was evaluated after five minutes of incubation. NaCl without bacteria was used as a negative control. HiB 770235 F+b+, fimbriated and encapsulated variant, (kindly provided by Loek van Alphen) was used as a positive control for hemagglutination. Experiments were independently repeated three times showing same results. Depicted are representative pictures for each strain.

S2

A

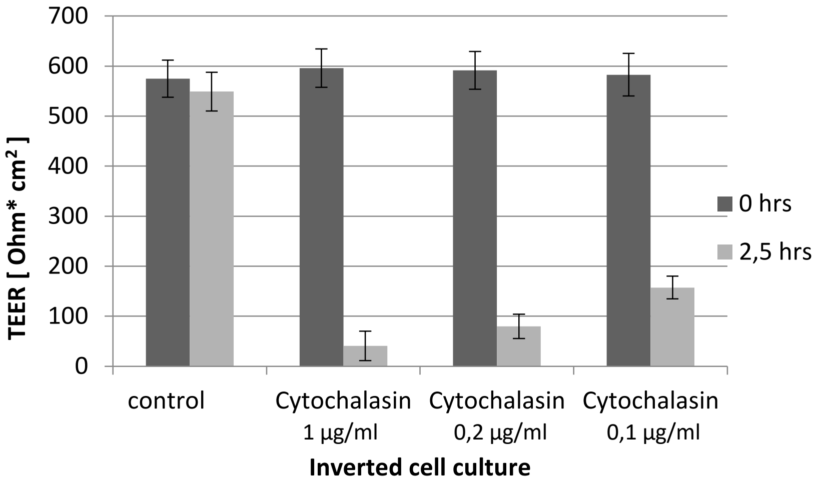

B

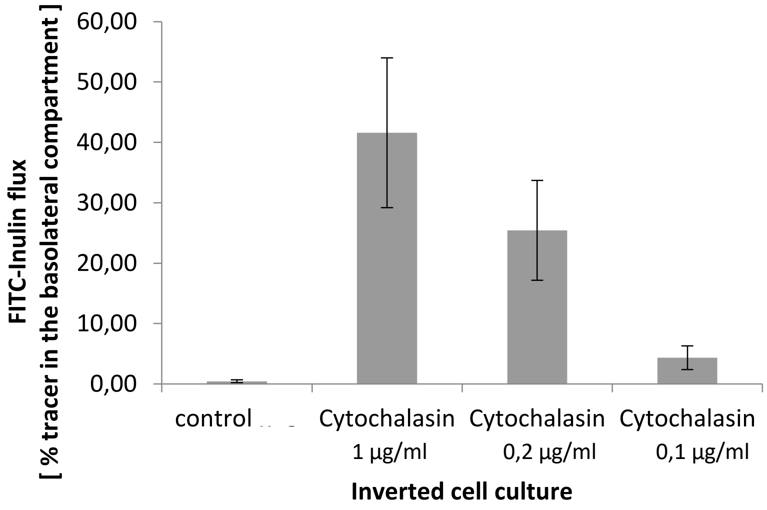

Figure S2: Cytochalasin D treatment impairs barrier function in a concentration dependent manner. HIBCPP cells were treated from the basolateral side with Cytochalasin D for 2,5 hrs in the inverted culture. (A) TEER values were measured before and after 2,5 hrs of Cytochalasin D treatment. (B) Permeability was monitored in parallel by FITC-labeld inulin flux. The experiment was performed with three filter inserts of inverted cultured HIBCPP cells with the following Cytochalasin D (Sigma) concentrations 1 µg/ml, 0,2 µg/ml and 0,1 µ/ml.
0 hrs TEER: Kruskal-Wallis test reveals p=0,9327, thus there is no difference between different treatments. 2,5 hrs TEER: Kruskal-Wallis test reveals p=0,0156, thus there is a significant difference between the treatments. Inulin: Kruskal-Wallis Test reveals p=0,0188, thus there is a significant difference between the treatments. In the two latter data sets a subsequent Wilcoxon 2 sample test is not possible due to the low sample size. The greatest differences in rank sums and medians, however, were observed between the untreated control and the highest Cytochalasin concentration of 1 µg/ml followed by 0,2 µg/ml and 0,1 µg/ml, which due to sample size, however, are not detectable.

S3

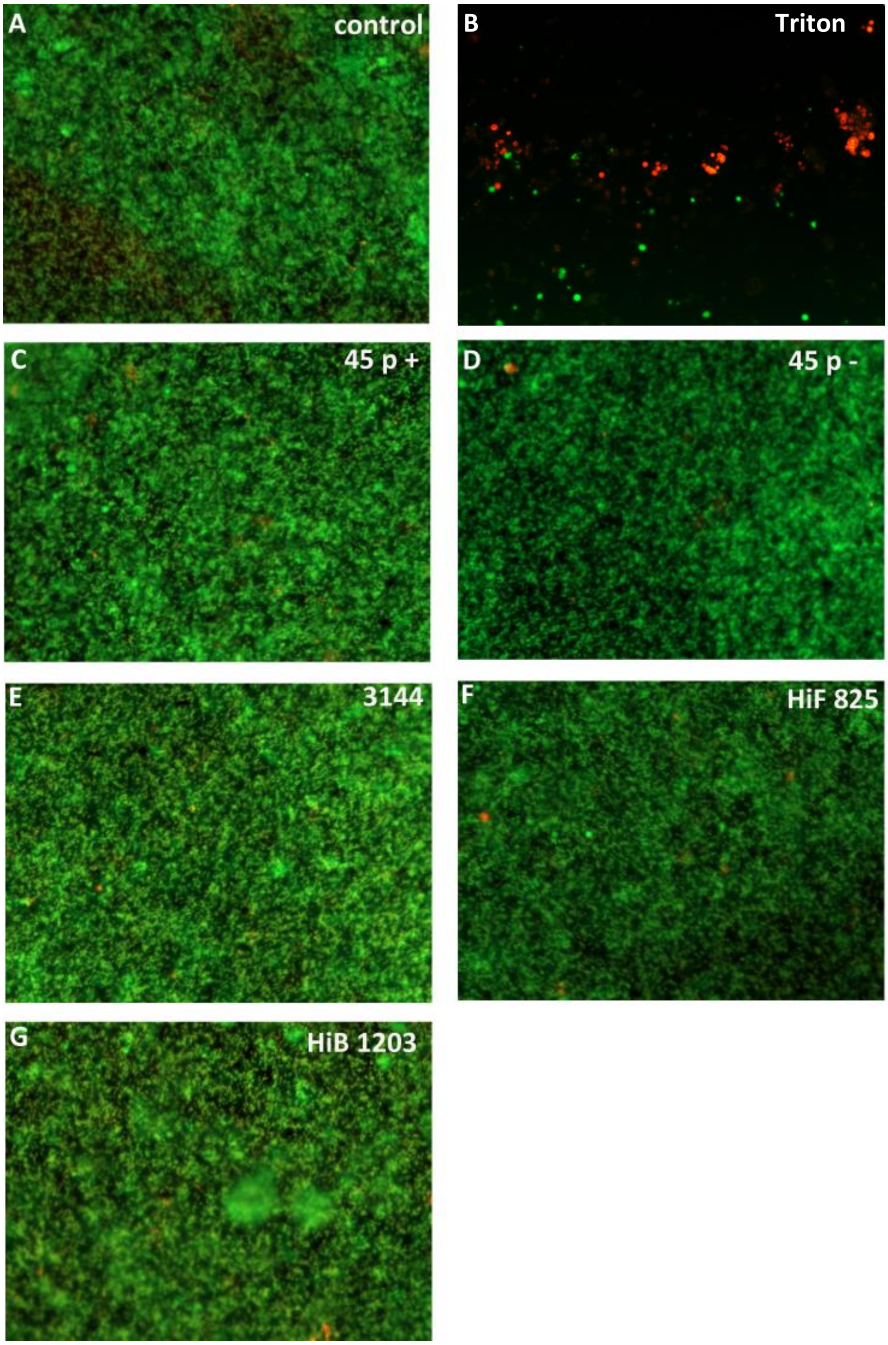

Figure S3: Cell viability of HIBCPP cells challenged with the indicated *H. influenzae* strains for 6 hrs or left untreated. Live dead staining (Thermo Fisher Scientific, Darmstadt, Germany) of confluent HIBCPP cells cultured in a 96 well plate corresponding to the standard cell culture where infection is performed from the apical cell side. Cells were stained according to the manufacturer's instructions and the results were documented by IF microscopy. Living cells are stained in green (due to the intracellular esterase activity), whereas dead cells are stained in red (due to the loss of the plasma membrane integrity and binding of ethidium homodimer to the DNA). Triton X-100 (10 % in PBS) treatment (Sigma) was used as a control affecting membrane permeability.

S4

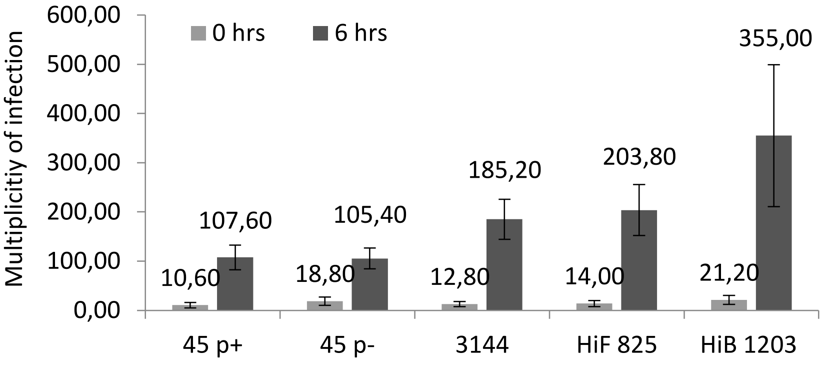

Figure S4: Corresponding growth curve (n=5) of the indicated *H. influenzae* strains, which were used for the calculation of the invasion and adhesion rates. Growth curves were performed in parallel to the infection experiments and invasion/adhesion numbers were referred to the corresponding bacterial numbers grown after 6 hrs of the corresponding experiment. Infection of standard and inverted cultures were carried out on the same day when TEER values were appropriate. Values show the Mean and standard deviation of the the Multiplicity of infection of HIBCPP cells on a single filter insert (~400.000 cells) at the time of infection and after 6 hrs of infection.
